# Predictive modeling for 14-day unplanned hospital readmission risk by using machine learning algorithms

**DOI:** 10.1186/s12911-021-01639-y

**Published:** 2021-10-20

**Authors:** Yu-Tai Lo, Jay Chiehen Liao, Mei-Hua Chen, Chia-Ming Chang, Cheng-Te Li

**Affiliations:** 1 Department of Geriatrics and Gerontology, National Cheng Kung University Hospital, College of Medicine, National Cheng Kung University, Tainan, Taiwan (R.O.C.); 2 Institute of Data Science, National Cheng Kung University, No. 1, University Road, Tainan City 701, Taiwan (R.O.C.); 3 Department of Medicine and Institute of Gerontology, College of Medicine, National Cheng Kung University, Tainan, Taiwan (R.O.C.)

**Keywords:** Unplanned readmission, Machine learning, Risk prediction model, Discharge planning, Healthcare quality indicators

## Abstract

**Background:**

Early unplanned hospital readmissions are associated with increased harm to patients, increased medical costs, and negative hospital reputation. With the identification of at-risk patients, a crucial step toward improving care, appropriate interventions can be adopted to prevent readmission. This study aimed to build machine learning models to predict 14-day unplanned readmissions.

**Methods:**

We conducted a retrospective cohort study on 37,091 consecutive hospitalized adult patients with 55,933 discharges between September 1, 2018, and August 31, 2019, in an 1193-bed university hospital. Patients who were aged < 20 years, were admitted for cancer-related treatment, participated in clinical trial, were discharged against medical advice, died during admission, or lived abroad were excluded. Predictors for analysis included 7 categories of variables extracted from hospital’s medical record dataset. In total, four machine learning algorithms, namely logistic regression, random forest, extreme gradient boosting, and categorical boosting, were used to build classifiers for prediction. The performance of prediction models for 14-day unplanned readmission risk was evaluated using precision, recall, F1-score, area under the receiver operating characteristic curve (AUROC), and area under the precision–recall curve (AUPRC).

**Results:**

In total, 24,722 patients were included for the analysis. The mean age of the cohort was 57.34 ± 18.13 years. The 14-day unplanned readmission rate was 1.22%. Among the 4 machine learning algorithms selected, Catboost had the best average performance in fivefold cross-validation (precision: 0.9377, recall: 0.5333, F1-score: 0.6780, AUROC: 0.9903, and AUPRC: 0.7515). After incorporating 21 most influential features in the Catboost model, its performance improved (precision: 0.9470, recall: 0.5600, F1-score: 0.7010, AUROC: 0.9909, and AUPRC: 0.7711).

**Conclusions:**

Our models reliably predicted 14-day unplanned readmissions and were explainable. They can be used to identify patients with a high risk of unplanned readmission based on influential features, particularly features related to diagnoses. The operation of the models with physiological indicators also corresponded to clinical experience and literature. Identifying patients at high risk with these models can enable early discharge planning and transitional care to prevent readmissions. Further studies should include additional features that may enable further sensitivity in identifying patients at a risk of early unplanned readmissions.

**Supplementary Information:**

The online version contains supplementary material available at 10.1186/s12911-021-01639-y.

## Introduction

Hospital readmissions disrupt the normality of the lives of families and caregivers of patients; moreover, they are associated with harm to patients, reduced quality of care [1], and increases in overall health care costs [2, 3]. The hospital readmission rate is considered a performance indicator to measure a hospital’s quality of care [4]. Furthermore, decreasing unnecessary hospital readmissions can potentially reduce financial and health care burden and improve the quality of care [5, 6].

One of the approaches for decreasing the hospital admission rate is to identify patients at risk of readmission; this will enable further investigations, and preventive strategies can then be developed because many readmissions are preventable [7, 8]. However, diverse and complex factors lead to readmissions, and clinicians cannot process all information to accurately identify at-risk patients [9]. Applying predictive models can direct medical attention toward patients with a high readmission risk, which leverages health care systems and saves health care expenditure.

Current models for readmission risk prediction include attributes describing patient’s initial admission; clinical data have been developed and validated for this, but they have yielded moderate discriminative ability [10, 11]. The complex interaction between readmission and potential risk makes accurate prediction of readmission difficult. Machine learning (ML) methods can harness high-dimensional medical data to generate accurate patient risk stratification models and shape health care decisions through the customization of care to individual patients [12].

Preliminary studies have demonstrated that for 30-day all-cause hospital readmission prediction, ML models are better than conventional predictive models [13, 14]. Nevertheless, only unplanned readmissions may lead to substandard care [15]. The likelihood of unplanned readmissions is the highest in the immediate postdischarge period [3], and early 14-day unplanned readmissions were demonstrated to be associated with quality of inpatient care; thus, they were deemed avoidable in cases of high-quality care [1]. Recent studies have shown that readmissions within the first 7 days of hospital discharge may be more preventable than later 30-day readmissions [7, 16, 17] and are mostly related to potential gaps in care during the index hospitalization [16, 18]. In Taiwan, the rate of unplanned 14-day readmission for the same or related diagnosis is among the continuous monitoring indicators of care quality of the National Health Insurance Administration; in turn, it affects the hospital accreditation and indirectly influences reimbursement to hospitals [19]. However, whether predictive models and significant predictors of 14-day unplanned hospital readmissions vary from those of 7-day or 30-day unplanned readmissions has not been thoroughly investigated.

Furthermore, predicting readmission early can improve the quality of care. Although ML has been successful with large datasets for predicting 30-day unplanned readmission [12, 20], studies investigating ML-based risk prediction models for identifying high-risk patients for 7- or 14-day unplanned hospital readmission are also lacking.

Therefore, the objective of our study was to build ML models that can accurately predict 14-day unplanned hospital readmissions and to identify influential risk factors in a cohort of patients discharged from a tertiary teaching hospital in Taiwan.

## Methods

### Study design and participants

This retrospective cohort study included consecutive patients discharged from a 1193-bed tertiary care academic medical center in Tainan, Taiwan, from September 1, 2018, to August 31, 2019. Patients who were aged < 20 years, who were admitted for cancer-related treatment, who participated in pharmaceutical clinical trial, who were discharged against medical advice, who died during admission, or who lived abroad were excluded from the study. The study protocol was approved by the institutional review board of the hospital (A-ER-108-309).

### Predictor variables

Data for analysis included 7 categories of variables extracted from hospital’s medical record dataset: (1) demographic characteristics; (2) health care utilization 6 months before index admission; (3) diagnoses 1 year before index admission including the total count of inpatient diagnoses in the past year (we collected 3 major diagnoses of each hospitalization; if the patient was admitted twice 1 year before the index admission, 6 inpatient diagnoses were collected), number of unique inpatient diagnoses in the past year (we deleted duplicate diagnoses from the total inpatient diagnoses), total counts of outpatient diagnoses in the past year, and the number of unique outpatient diagnoses in the past year; (4) overall comorbidity and functional evaluation on index admission including Charlson comorbidity index [21], presence of depression according to International classification of Diseases, Tenth Revision code [22], nutrition status according to Malnutrition Universal Screening Tool [23], and mood status according to Brief Symptom Rating Scale [24]; (5) health care services–related variables during index admission; (6) one-time laboratory values recorded just before discharge; (7) discharge-related variables. The detailed descriptions of all predictor variables are listed in Table 1.

**Table 1:** List of variables and their corresponding category utilized in predicting 14-day unplanned readmission risk

Category	Variable
Demographic	Age; Sex; Marital status; Religion; Education; Area of residence; Living alone
Health care utilization 6 months before index admission	Number of hospitalizations; Emergency department visits; Outpatient visits
Diagnoses 1 year before index admission	The total count of inpatient diagnoses; Number of unique inpatient diagnoses; Total counts of outpatient diagnoses; The number of unique outpatient diagnoses
Overall comorbidity and functional evaluation on index admission	The 3 major diagnoses of index admission; Charlson comorbidity index; Depression diagnoses; Consciousness level; Activities of daily living according to dependency level in mobility, dressing, feeding, toileting, and bathing; Nutrition status; Mood; Urinary incontinence; History of fall
Health care services–related variables during index admission	Index type of admission; Disease-Related Group of the index admission; Health education
One-time laboratory values recorded just before discharge	Hematocrit; White blood cell count; Red blood cell count; Mean corpuscular volume; Platelet count; Hemoglobin; Prothrombin time; Blood Urea Nitrogen; Creatinine; Aspartate Aminotransferase; Alanine Aminotransferase; Lactate Dehydrogenase; γ-glutamyl transferase; Total Bilirubin; Potassium; Calcium; Sodium; Albumin, C-reactive protein; Thyroid-Stimulating Hormone
Discharge-related factors	Registered in the discharge planning services; Vital signs recorded 24 h before discharge (systolic and diastolic blood pressure, pulse rate, respiratory rate, and body temperature); Department of discharge; Attending physician’s employee identity and years of experience; Number of discharge medication categories; Total number of tablets in discharge medication; Discharge destination; Discharge with pressure injury (or injuries); Types of catheters at discharge; Index hospital length of stay

### Validation of hospital data

All variables that constitute the data for analysis were validated through the medical record review of randomly identified patient records at a 1:50 proportion for participants had 14-day unplanned readmission by one of the authors (MHC).

### Preprocessing of features

We grouped certain categories together to reduce the numbers of categories of these features. Assuming that missing values are distributed randomly, we used imputation, which is a common approach for dealing with missing values [25]. Missing values in continuous features were filled with the median values of the features. We did not fill them with the mean values due to the asymmetric distribution of features. As for categorical features, we filled missing values with modes if they had a relatively smaller proportion. Otherwise, we treated missing values as a new category. For binary features, we filled missing values with a negative value.

### Study outcome

The primary outcome was unplanned readmissions within 14 days of discharge after index admission. Unplanned readmission was defined as admission for the same or a related diagnosis according to the National Health Insurance Administration's definition. After thoroughly evaluating readmitted patients' diagnoses for readmission and their clinical courses, the attending physicians in charge of the readmissions made the distinction of unplanned readmissions.

### ML techniques

We used 4 ML algorithms, namely logistic regression [26], random forest (RF) [27], extreme gradient boosting (Xgboost) [28], and gradient boosting with categorical features support (Catboost) [29], to build classifiers for prediction. Logistic regression is a traditional statistical model and usually used to be the baseline to compare to ML models [13]. RF, Xgboost, and Catboost have shown acceptable performance in predicting unplanned readmissions in previous studies [30].

To address the assumption of non-collinearity for logistic regression, we computed Variance Inflation Factor (VIF) values to detect if collinearity existed and removed features with collinearity. First, we computed VIF values for each feature. Then we removed the feature with the highest VIF value and compute VIF values again and repeated this procedure until all VIF values are smaller than 4.

Feature importance can be obtained with RF, Xgboost, and Catboost models based on their use of features during training. Feature importance demonstrates how much the prediction changes as the feature values vary. Higher feature importance indicates the higher importance of the feature to the model prediction. Through feature selection based on feature importance, we selected the most influential features to enhance the model’s generalizability and performance and make the model practically usable. We set several thresholds of feature importance and chose values that provided an appropriate number of features the model.

Models were trained using Python 3.6.9 on a Linux Intel Xenon Gold 6138 processor with 2.0 GHz RAM and a 450G CPU. An NVIDIA Tesla V100 32 GB GPU was used to speed up the training process for Catboost models. We implemented models with Scikit-Learn, Xgboost, and Catboost packages of Python. Model hyperparameters not learnable during training and determined the structure of models, were set as the defaulted values of these packages.

### Training and evaluation

The dataset was split into training (75%) and testing (25%) sets with stratified random sampling to fix the proportion of patients with unplanned readmission in both subsets. The predictive models for readmission were trained on the training set and were applied and evaluated on the testing set. The fivefold cross-validation approach was used to obtain reliable results for evaluating prediction models or for obtaining reliable results. The original training set was split into 5 folds through stratified random sampling. For the *i*th iteration, fold *i* was treated as the validation set and the remaining 4 folds were used to train the model. The model was evaluated using the validation set. The procedure was repeated for 5 iterations. Evaluation results of 5 iterations were collected to compute the mean value and standard deviation.

We used 5 commonly used evaluation indices to evaluate the models, namely precision score (positive predict rate), recall score (sensitivity), F1-score, area under the receiver operating characteristic curve (AUROC), and area under the precision–recall curve (AUPRC). These evaluation indices are commonly reported in the evaluation of classification problems with ML [31]. Furthermore, AUPRC is appropriate for prediction tasks with a low rate of positive cases [32]. Their definitions are listed in Table 2.

**Table 2:** Definitions of evaluation metrics

Notation/evaluation index	Description/definition
*TP*	*True positive*. The number of patients who had unplanned readmission and were also predicted to have unplanned readmission by the model
*FP*	*False positive*. The number of patients who did not have unplanned readmission but were predicted to have unplanned readmission by the model
*TN*	*True negative*. The number of patients who did not have unplanned readmission and were also not predicted to have unplanned readmission by the model
*FN*	*False negative*. The number of patients who had unplanned readmission but were not predicted to have unplanned readmission by the model
*Precision*	*TP*/(*TP* + *FP*)
*Recall*	*TP*/(*TP* + *FN*)
*F1-score*	The harmonic mean of *precision* and *recall*. The formula is as follows: *F1* = 2 / (1 / *Precision* + 1 / *Recall*)
*AUROC*	Area under the receiver operating characteristic curve
*AUPRC*	Area under the precision–recall curve

### Model interpretation

We adapted SHapley Additive exPlanations (SHAP) [33], a game theory–based framework with feature importance calculation, to interpret our ML model. It assigns an importance value (SHAP value) to each feature to explain the predication of each observation. It can also summarize how every feature contribute to the prediction. To calculate SHAP values on categorical features, the approach to turn every category in a categorical feature into dummy variables, namely “one-hot encoding”, is utilized naturally.

## Results

### Cohort characteristics

**Fig. 1:**
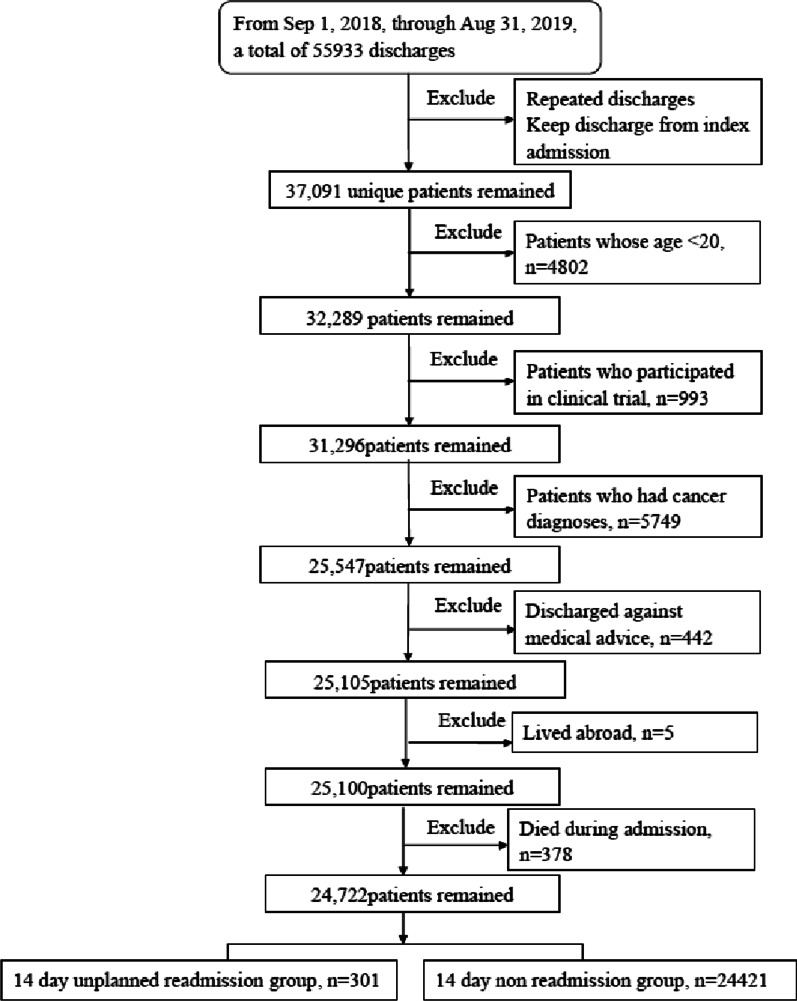
Flowchart of study cohort selection

From September 1, 2018, to August 31, 2019, a total of 37, 091 adult patients were discharged, with a total of 55, 933 discharges (including repeated admissions). Of these, 530 patients had unplanned readmission and 31, 759 patients had no unplanned readmission (unplanned readmission rate: 1.64%). After exclusion, 301 and 24, 421 patients with and without unplanned readmission were included (unplanned readmission rate: 1.22%). The mean age of the cohort was 57.34 ± 18.13 years. The training and validation cohorts consisted of 24, 722 patients (Fig. 1).

Characteristics, including laboratory values, between patients with and without unplanned readmission are presented in Additional file 1.

### ML model performance

**Fig. 2:**
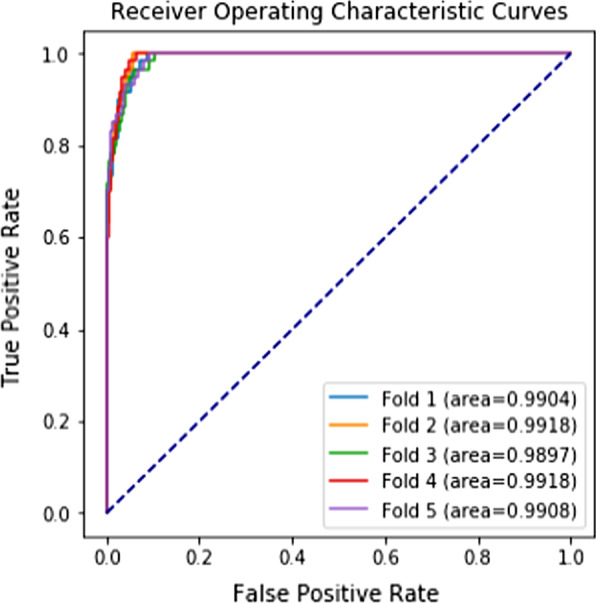
Receiver operating characteristic curves of Catboost with 21 features

**Fig. 3:**
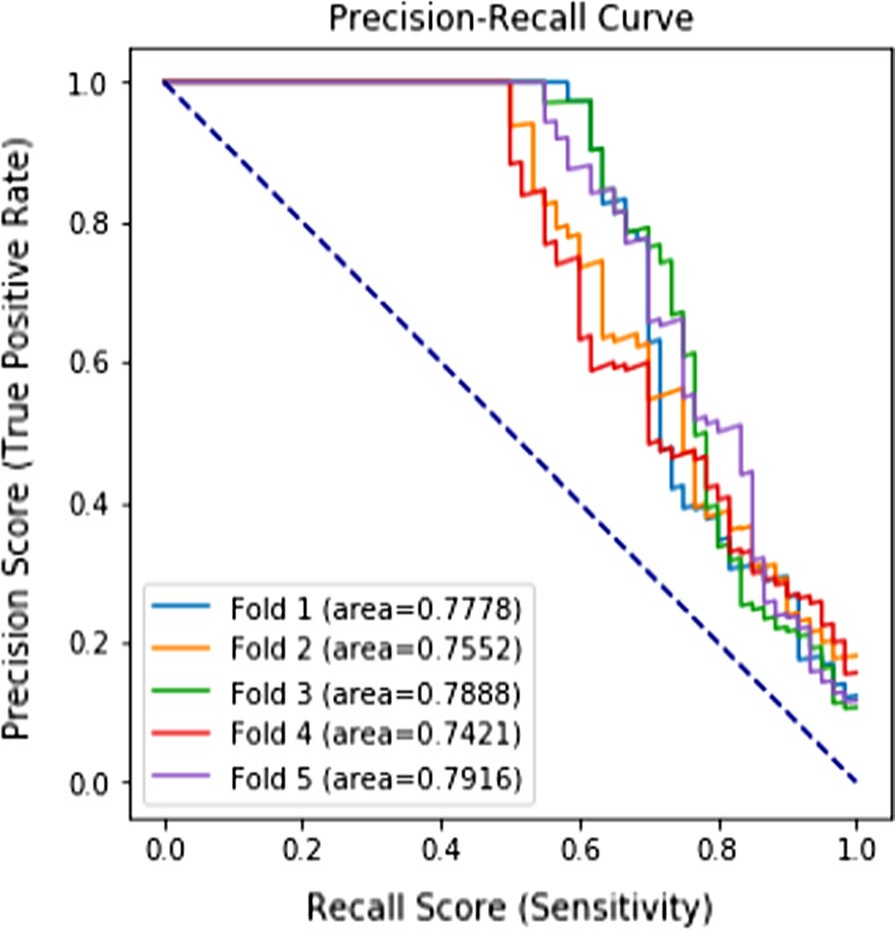
Precision–Recall Curves of Catboost with 21 features

By computing VIF values, we found that collinearity existed in the 70 original features. We remained 27 features without collinearity (VIF < 4) and created a logistic regression model with these predictors (Additional file 2). Among the 4 ML algorithms, the logistic regression model had the worst performance, and Catboost had the best performance. Table 3 lists the performance results of models evaluated on the testing set and the different performance results of Catboost models during the feature selection process. After feature selection, the Catboost model with 21 features (Catboost 4 in Table 3) performed the best in terms of AUROC (0.9909) and AUPRC (0.7711), which considered both positive rate and sensitivity. If more features were removed (Catboost 5 and 6 in Table 3), the precision score decreased. Therefore, we adopted Catboost 4 with 21 features as the final model. Figures 2 and 3 present its receiver operating characteristic curve and precision–recall curve.

**Table 3:** Performance metrics of the LACE model and machine learning models based on the testing set with fivefold cross-validation (Mean ± Standard Deviation, Unit: %)

Model (#Features)	Precision	Recall	F1-Score	AUROC	AUPRC
LACE (4)	2.97 ± 0.15	68.67 ± 3.86	5.70 ± 0.29	70.58 ± 1.88	34.63 ± 0.00
Logistic Regression: original features (70)	45.76 ± 15.72	4.00 ± 2.00	7.35 ± 3.59	80.46 ± 2.43	10.26 ± 2.23
Logistic Regression: original features (27)	43.62 ± 20.73	5.00 ± 1.05	8.84 ± 2.00	82.88 ± 3.57	11.66 ± 3.54
Random Forest: original features (70)	100.00 ± 0.00	41.33 ± 3.86	58.39 ± 3.79	97.89 ± 0.71	70.15 ± 4.23
Xgboost: original features (70)	93.23 ± 5.35	45.67 ± 3.89	61.25 ± 4.32	97.95 ± 0.52	66.52 ± 2.23
Catboost 1 (C1): original features (70)	93.77 ± 4.05	53.33 ± 5.27	67.80 ± 4.47	99.03 ± 0.07	75.15 ± 1.92
Catboost 2: features in C1 with importance > 0.5 (35)	95.12 ± 2.54	56.00 ± 5.33	70.29 ± 3.84	99.04 ± 0.09	76.11 ± 2.45
Catboost 3: features in C1 with importance > 0.6 (28)	95.09 ± 3.09	55.33 ± 5.31	69.74 ± 3.99	99.08 ± 0.08	76.69 ± 1.85
Catboost 4: features in C1 with importance > 0.8 (21)	94.70 ± 3.52	56.00 ± 6.02	70.10 ± 4.40	99.09 ± 0.08	77.11 ± 1.93
Catboost 5: features in C1 with importance > 0.9 (19)	93.20 ± 1.59	55.33 ± 5.72	69.29 ± 4.76	99.07 ± 0.10	76.80 ± 1.64
Catboost 6: features in C1 with importance > 1.1 (14)	91.46 ± 2.12	56.67 ± 4.47	69.86 ± 3.51	99.00 ± 0.11	76.97 ± 2.90

AUROC = area under the receiver operating characteristic curve; AUPRC = area under the precision–recall curve

### Significant predictors

**Fig. 4:**
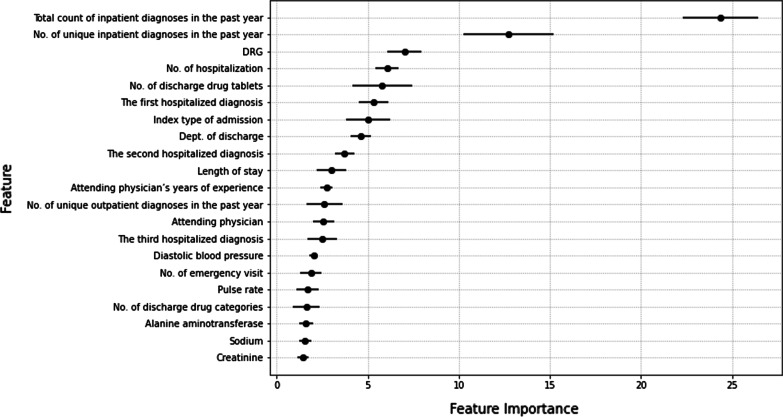
Feature importance in Catboost with 21 features

The final Catboost model has 21 useful features. Figure 4 shows the importance of these features with the average value and standard deviation in 5-fold cross-validation.

### Model interpretation

**Fig. 5:**
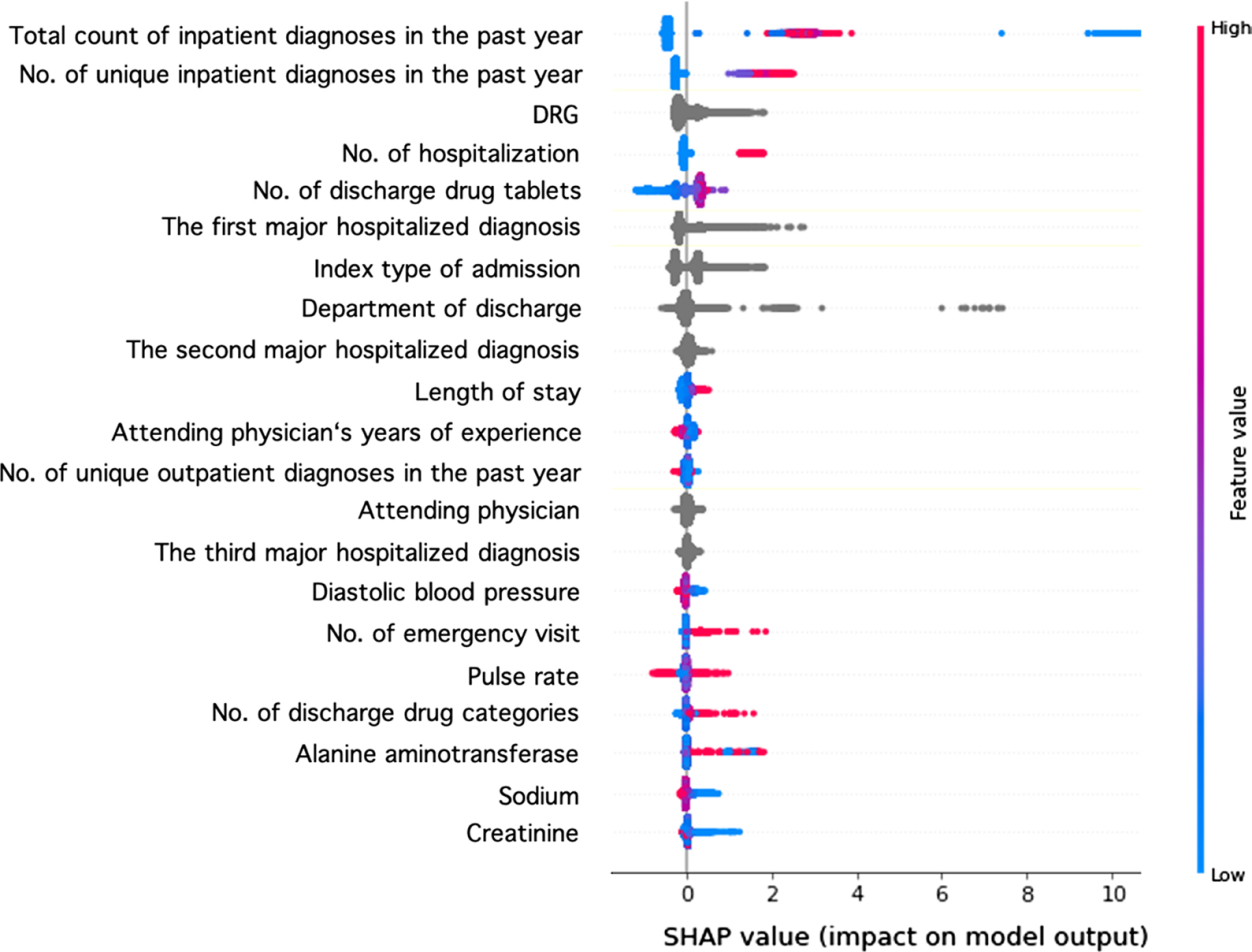
Association between feature value and SHAP value in Catboost with 21 features

**Fig. 6:**
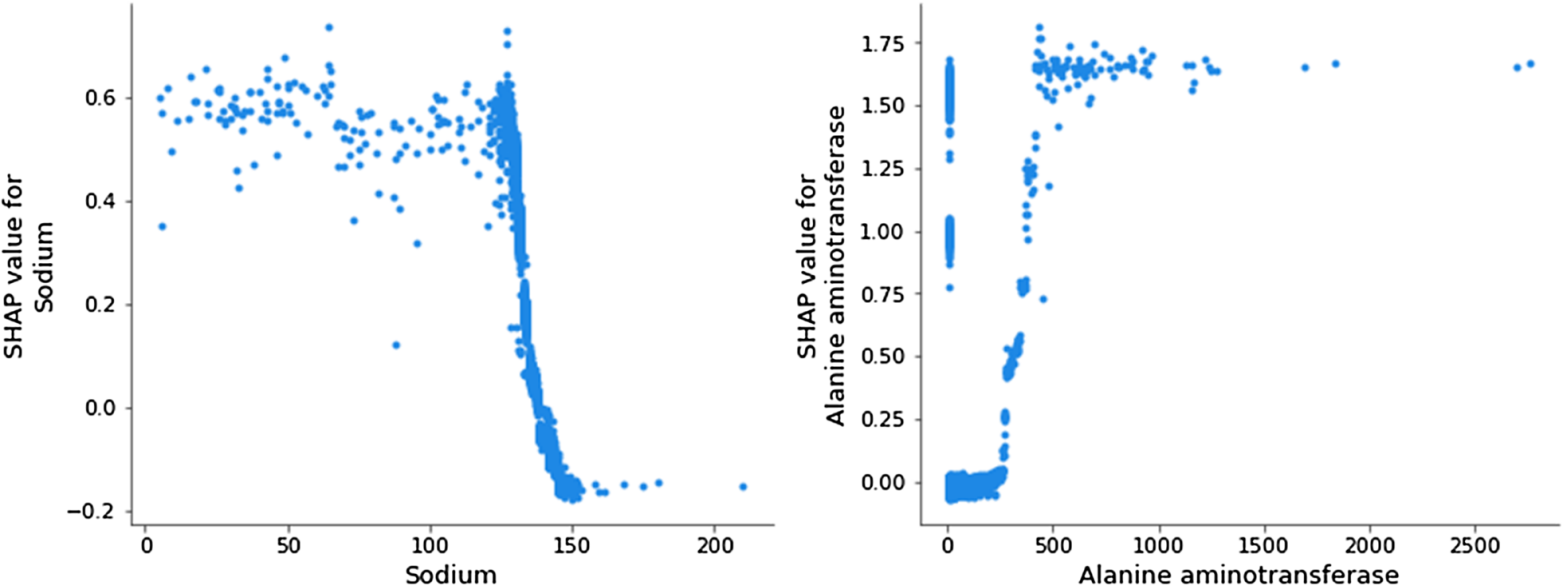
Association of SHAP value with Sodium (Left) and Alanine aminotransferase (Right) in Catboost with 21 features

In Figs. 5 and 6, SHAP values are used to demonstrate how our Catboost model operates to classify patients as cases with 21 features. Among training sets in 5 folds, we randomly take one to demonstrate.

In Fig. 5, red points refer relatively higher values and blue points refer relatively lower values in numerical features. For categorical features, one grey point represents a category in the given feature. And its location on the horizontal axis indicates the SHAP value that patients who belong to this category would be assigned from this feature. Overall, the model yielded a higher probability of unplanned readmission to patients with more inpatient diagnoses or higher numbers of unique inpatient diagnoses 1 year before index admission, indicating that these patients are more likely to have unplanned readmissions. In addition, the distribution of grey points of feature “DRG” is wider than that of feature “Attending physician”, meaning that the variation of SHAP values of different DRGs is higher than that of different attending physicians. That is, compared to different attending physicians, different DRGs may contribute larger difference of unplanned readmission possibility. This corresponds to Fig. 4, which shows that the average feature importance of “DRG” is higher than that of “Attending physician” and that their bars of one standard deviation even do not overlap (7.02 ± 0.93 vs. 2.57 ± 0.58). Figure 6 shows the association of SHAP values with values of 2 physiological features, namely sodium and alanine aminotransferase.

## Discussion

Although recent studies have used ML to predict 30-day all-cause or unplanned readmission risk, analyses for predicting 14-day unplanned readmission remain rare in the literature. To the best of our knowledge, this is the first study to use ML to predict 14-day unplanned readmission and to select features to establish prediction models and the first study to use ML for predicting unplanned readmissions by using local data in Taiwan.

Accurately identifying patients at risk of unplanned readmission shortly after discharge can enable early discharge planning and transitional care to prevent recurrent readmissions. Our findings demonstrated that ML algorithms can predict a patient’s risk of 14-day unplanned readmission with good discrimination and precision. They also suggested that unlike conventional approaches such as logistic regression, other ML algorithms have the advantage of convenient utility besides accurate prediction. That is, we do not need to consider collinearity of predictors when fitting an ML model such as Catboost.

The most important finding is that the final ML model demonstrated good discrimination (AUROC > 0.99 and AUPRC > 0.77) with excellent precision (0.9470) and moderate sensitivity (0.5600). Furthermore, we computed the LACE score (calculated at discharge using 4 items: length of stay, acuity of admission, comorbidities and emergent department visits 6 months before index admission) [34], a well-known readmission risk assessment score [34], in our cohort. Although LACE had a relatively high sensitivity (0.6867), its precision score was extremely low. Our final ML model strongly outperformed LACE in terms of evaluation metrics except for the recall score (precision: 0.9470 vs 0.0297, F1-score: 0.7010 vs 0.0570, AUROC: 0.9909 vs 0.7058, and AUPRC: 0.7711 vs 0.3463). Besides LACE, according to previous reports, our ML model seemed to outperform other well-known developed readmission risk assessment scores, such as PARR-30 [35] (calculated with age, place of residence, acuity of admission, emergent department visits in the last year, history in the prior two years of 11 major health conditions drawn from the Charlson co-morbidity index, and the hospital of admission), and HOSPITAL score [36] (calculated with hemoglobin, discharge from an oncology service, sodium level, procedure during the index admission, index type of admission, number of admissions during the last year, and length of stay). The performance of PARR-30 and HOSPITAL were AUROC: PARR-30 = 0.7, HOSPITAL: 0.72; precision: PARR-30 = 0.59; sensitivity: PARR-30 = 0.054). The benefit of ML is that it is trained for each hospital and weighted for individual characteristics. Furthermore, several studies have demonstrated that ML models are better than conventional models for all-cause hospital readmission prediction [13, 14, 30, 37, 38], and some studies have evaluated models for unplanned admissions [12, 20, 39]. Goyal et al. used a national database of 59, 145 patients who underwent spinal fusion to evaluate seven ML algorithms, and all models showed moderate performance with 30-day unplanned readmission (AUROC: 0.63–0.66, sensitivity: 0.46–0.64, and precision: 0.07). Among the seven ML models, gradient boosting machines performed the best [12]. Morgan et al. compared ML with conventional risk prediction scores for 30-day unplanned readmissions in 14,062 patients at 3 different hospitals, and ML score predicted readmissions better than conventional scores (AUROC: 0.81, precision: 0.375, and sensitivity: 0.283) [20]. Considering overall performance, our final model seems to have satisfying prediction precision and sensitivity. A likely explanation for the satisfying performance of ML in our study is that we adopted Catboost, a ML algorithm designed for processing categorical data. Among 21 features in the final model, 6 of them are categorical features. Furthermore, the numbers of categories of features were extremely large in our data (e.g., 684 in DRG and 297 in attending physicians’ employee identity), which may be difficult for non-Catboost models to deal with.

The second important finding is that our ML prediction model successfully identified several useful predictors, which have also been used in the conventional risk assessment scores, such as LACE, PARR-30, and HOSPITAL. In the present model, patient age and place of residence were not associated with readmission risk, but hospitalizations 6 months before index admission was an important feature. This finding is consistent with the results of an updated systemic review regarding prediction models of 28- or 30-day unplanned hospital readmissions, which showed that the number of previous admissions ranks the fourth among top 10 most important variables and is included in 29 unplanned readmission prediction models [10]. Furthermore, the number of total discharge medication tablets and medication categories were influential features in our model. Prescribed drug–related readmissions represent a nonnegligible proportion of readmissions, particularly among older patients [30, 40, 41]. Our study did not include high-risk medications as a predictor variable, and future investigation is warranted to understand the effect of different medications on 14-day readmission risk.

The strength of this study is that it includes multiple predictor variables from demographic characteristics, prior health care utilization, diagnosis-related variables, overall health and function assessment on the index admission, variables related to healthcare services during admission, laboratory tests on discharge, and discharge-related variables for analysis. Among the 21 features in the final model, most of the predictors could be identified at an early stage of admission; therefore, these variables can be used to estimate the probability of readmission soon after patients are admitted [37]. Other variables may not be modified by actions taken at discharge, but the most effective interventions preventing readmission in fact are related to postdischarge support to patients and caregivers [42]. By using the prediction model, patients identified as at-risk can be closely monitored and early outpatient follow-up or referrals to home health care services can be arranged.

This study has several limitations. First, data were retrospectively extracted from medical records, which may have reduced our ability to identify all risk factors for readmission. Second, we did not consider readmission to another facility because the data were limited to readmissions in the same hospital. Third, this study involved patients of a single academic tertiary hospital, and our findings may not be generalizable to other facilities; hence, further external validation is required. Finally, we used cross-sectional features; we lacked features with sequential or temporal trajectory of events in electronic health records over time, which contains important information about disease progression and patient status. Access to large volumes of patient records with a sequential trajectory of events, such as electronic health records, warrants further investigation to improve prediction sensitivity and performance. Nevertheless, our findings could lay the groundwork for future studies using ML as a risk stratification tool for early unplanned readmissions.

## Conclusions

ML prediction models can help clinicians to accurately identify patients likely to experience early unplanned readmission. Our study results enable clinicians to identify patients at a high risk of hospital readmission and also suggest interventions that can be initiated during hospitalization, such as providing adequate patient or family education before discharge. Although our work has scope for improvement, we believe that it has set the stage for further research to improve the accuracy of predicting early readmission risk.

## Supplementary Information

**Additional file 1**. Numbers and proportions of missing values in study variables.

**Additional file 2**. The Variance Inflation Factor Values of 27 Features Included in the Final Logistic Regression Model.

## Abbreviations

ML: Machine learning
DRG: Disease-related group
RF: Random forest
Xgboost: Extreme gradient boosting
Catboost: Gradient boosting with categorical features
AUROC: Area under the receiver operating characteristic curve
AUPRC: Area under the precision–recall curve
SHAP: SHapley additive exPlanations

## Declarations

### Availability of data and materials

Data and resources will be shared with other eligible investigators through academically established means. The datasets used and analyzed during the study will be available from the corresponding author on reasonable request.
