# Correction to: Predictive modeling for 14-day unplanned hospital readmission risk by using machine learning algorithms

**DOI:** 10.1186/s12911-022-01804-x

**Published:** 2022-03-25

**Authors:** Yu-Tai Lo, Jay Chiehen Liao, Mei-Hua Chen, Chia-Ming Chang, Cheng-Te Li

**Affiliations:** 1 Department of Geriatrics and Gerontology, National Cheng Kung University Hospital, College of Medicine, National Cheng Kung University, Tainan, Taiwan (R.O.C.); 2 Institute of Data Science, National Cheng Kung University, No. 1, University Road, Tainan City, 701 Taiwan (R.O.C.); 3 Department of Medicine and Institute of Gerontology, College of Medicine, National Cheng Kung University, Tainan, Taiwan (R.O.C.)

## Correction to: BMC Med Inform Decis Mak (2021) 21:288 10.1186/s12911-021-01639-y

Following publication of the original article [1], the following errors were reported:

1. Jah Chiehen Liao’s name was misspelled as ‘Jay Chie-hen Liao’
2. A note marking Yu-Tai Lo and Jay Chiehen Liao as co-first authors was missing
3. A grant number was missing in the Acknowledgement declaration

The correct authorship list is given in this Correction article and the corrected Acknowledgement declaration is given below, with the missing number in bold:

**Acknowledgements**

We thank the nursing supervisor of discharge planning Ms. Hsiu-Hua Lee, discharge planning nurses, and the information technicians at National Cheng Kung University Hospital for helping us collect data from patients’ medical records. This work is supported by the Ministry of Science and Technology (MOST) of Taiwan under grants 109-2636-E-006-017 (MOST Young Scholar Fellowship), 110-2221-E-006-001, 110-2221-E-006-136-MY3, and **110-2634-F-002-051**.

The original article [1] has been updated.
