# Supplementary material for: Predictive modeling for 14-day unplanned hospital readmission risk by using machine learning algorithms
Source: BMC Med Inform Decis Mak. 2021 Oct 20;21:288. doi: 10.1186/s12911-021-01639-y (PMC8527795; doi:10.1186/s12911-021-01639-y)
Supplement: Supplementary file 1 — Additional file 1. Numbers and proportions of missing values in study variables. [file 12911_2021_1639_MOESM1_ESM.docx]

**Additional file 1. Numbers and Proportions of Missing Values in Study Variables**

| Variable | Non unplanned readmission (n=24421) | | Unplanned readmission (n=301) | | |
| --- | --- | --- | --- | --- | --- |
|  |  | No. (%) of  Missing Values |  | No. (%) of  Missing Values | |
| **Socio-Demographic** |  |  |  |  | |
| Age, mean (SD) | 57.25 (18.11) | 0 (0.00) | 64.90 (17.74) | 0 (0.00) | |
| Gender, No. (% ) |  | 0 (0.00) |  | 0 (0.00) | |
| Female | 12846 (52.60) |  | 136 (45.18) |  | |
| Male | 11575 (47.40) |  | 165 (54.82) |  | |
| Education, No. (% ) |  |  |  |  | |
| illiterate or Kindergarten | 1960 (8.03) |  | 35 (11.63) |  | |
| Primary school | 5325 (21.81) |  | 93 (30.90) |  | |
| Junior high school | 3648 (14.94) |  | 44 (14.61) |  | |
| Senior high school | 5706 (23.37) |  | 73 (24.25) |  | |
| college/University | 6500 (26.62) |  | 46 (15.28) |  | |
| Postgrauduate and above | 1282 (5.25) |  | 10 (3.32) |  | |
| Marrital status, No. (% ) |  |  |  |  | |
| Separate | 48 (0.20) |  | 0 (0.00) |  | |
| Have partner | 46 (0.19) |  | 2 (0.66) |  | |
| Widowed | 1966 (8.05) |  | 37 (12.29) |  | |
| Married | 16878 (69.11) |  | 213 (70.76) |  | |
| Single | 3839 (15.72) |  | 30 (9.97) |  | |
| Divorced | 960 (3.93) |  | 13 (4.32) |  | |
| Missing | 684 (2.80) |  | 6 (1.99) |  | |
| Religion, No. (% ) |  |  |  |  | |
| Folk belief | 10065 (41.21) |  | 137 (45.51) |  | |
| Taosim | 4442 (18.19) |  | 53 (17.61) |  | |
| Buddhism | 2617 (10.72) |  | 27 (8.97) |  | |
| Christian | 1022 (4.18) |  | 11 (3.65) |  | |
| Catholics | 133 (0.54) |  | 3 (1.00) |  | |
| Others | 126 (0.52) |  | 1 (0.33) |  | |
| Nil | 5494 (22.50) |  | 65 (21.59) |  | |
| Missing | 522 (2.14) |  | 4 (1.33) |  | |
| Area of residence, No. (% ) |  | 0 (0.00) |  | 0 (0.00) | |
| Urban | 19457 (79.67) |  | 240 (79.73) |  | |
| Rural | 4964 (20.33) |  | 61 (20.27) |  | |
| No. of emergency visit, mean (SD) | 0.73 (0.92) | 0 (0.00) | 1.98 (2.22) | 0 (0.00) | |
| No. of outpatient visit, mean (SD) | 4.22 (5.08) | 0 (0.00) | 7.46 (8.17) | 0 (0.00) | |
| No. of hospitalization, mean (SD) | 0.09 (0.49) | 0 (0.00) | 0.97 (1.39) | 0 (0.00) | |
| Total count of inpatient diagnoses, mean (SD) | 11.72 (30.97) | 0 (0.0) | 65.61 (92.46) | 0 (0.0) | |
| Number of unique inpatient diagnoses, mean (SD) | 1.42 (1.33) | 0 (0.0) | 3.82 (3.17) | 0 (0.0) | |
| Total counts of outpatient diagnoses, mean (SD) | 231.23 (411.23) | 0 (0.0) | 469.68 (530.14) | 0 (0.0) | |
| Number of unique outpatient diagnoses, mean (SD) | 5.26 (4.17) | 0 (0.0) | 8.03 (5.69) | 0 (0.0) | |
| Charlson comorbidity index, mean (SD) | 0.55 (1.15) | 0 (0.0) | 1.07 (2.07) | 0 (0.0) | |
| Diagnosis of depression, No. (% ) | 0 (0.0) |  | 0 (0.0) |  | |
| Yes | 907 (3.71) |  | 11 (3.65) |  | |
| No | 23514 (96.29) |  | 290 (96.35) |  | |
| Nutrition (MUST) , mean (SD) | 0.35 (0.77) | 542 (2.22) | 0.67 (1.10) | 7 (2.28) | |
| Mood (BSRS) , mean (SD) | 0.86 (0.96) | 4481 (18.35) | 0.79 (0.81) | 78 (25.91) | |
| Eye opening, No. (% ) |  |  |  |  | |
| No opening | 177 (0.72) |  | 8 (2.66) | |  |
| Opening in response to pain | 165 (0.68) |  | 5 (1.66) | |  |
| Opening in response to speech | 300 (1.23) |  | 16 (5.32) | |  |
| Eyes opening spontaneously | 23226 (95.11) |  | 267 (88.70) | |  |
| Missing | 553 (2.26) |  | 5 (1.66) | |  |
| Verbal response, No. (% ) |  |  |  | |  |
| No verbal response | 230 (0.94) |  | 9 (2.99) | |  |
| Incomprehensible sounds. | 232 (0.95) |  | 18 (5.98) | |  |
| Inappropriate words | 148 (0.61) |  | 3 (1.00) | |  |
| Confused | 699 (2.86) |  | 21 (6.98) | |  |
| Oriented | 21945 (89.86) |  | 226 (75.08) | |  |
| Not testable (aphasia) | 83 (0.34) |  | 1 (0.33) | |  |
| Not testable (intubation) | 462 (1.89) |  | 12 (3.99) | |  |
| Not testable (tracheostomy) | 109 (0.45) |  | 7 (2.33) | |  |
| Missing | 513 (2.10) |  | 4 (1.33) | |  |
| Motor response, No. (% ) |  |  |  | |  |
| No motor response | 114 (0.47) |  | 4 (1.33) | |  |
| Abnormal extension | 36 (0.15) |  | 3 (1.00) | |  |
| Abnormal Flexion | 88 (0.36) |  | 7 (2.33) | |  |
| Normal Flexion | 307 (1.26) |  | 18 (5.98) | |  |
| Localizes to pain | 576 (2.36) |  | 21 (6.98) | |  |
| Obeys commands | 22652 (92.76) |  | 243 (80.73) | |  |
| Missing | 648 (2.65) |  | 5 (1.66) | |  |
| Mobility, No. (% ) |  |  |  | |  |
| Independent | 837 (3.43) |  | 17 (5.65) | |  |
| Walk with help | 3484 (14.27) |  | 66 (21.93) | |  |
| Immobile | 1347 (5.52) |  | 58 (19.27) | |  |
| Missing | 18753 (76.79) |  | 160 (53.16) | |  |
| Activity of daily living: feeding, No. (% ) |  |  |  | |  |
| Independent | 140 (0.57) |  | 2 (0.66) | |  |
| Needs help | 2349 (9.62) |  | 39 (12.96) | |  |
| Unable | 1303 (5.34) |  | 58 (19.27) | |  |
| Missing | 20611 (84.40) |  | 202 (67.11) | |  |
| Activity of daily living: dressing, No. (% ) |  |  |  | |  |
| Independent | 135 (0.55) |  | 1 (0.33) | |  |
| Needs help | 2349 (9.62) |  | 49 (16.28) | |  |
| Unable | 1326 (5.43) |  | 58 (19.27) | |  |
| Missing | 20611 (84.40) |  | 193 (64.12) | |  |
| Activity of daily living: Toilet use, No. (% ) |  |  |  | |  |
| Independent | 142 (0.58) |  | 1 (0.33) | |  |
| Needs help | 2757 (11.29) |  | 56 (18.60) | |  |
| Unable | 1329 (5.44) |  | 58 (19.27) | |  |
| Missing | 20193 (82.69) |  | 186 (61.79) | |  |
| Activity of daily living: Bathing, No. (% ) |  |  |  | |  |
| Independent | 18497 (75.74) |  | 158 (52.49) | |  |
| Needs help | 2552 (10.45) |  | 50 (16.61) | |  |
| Unable | 1319 (5.40) |  | 57 (18.94) | |  |
| Missing | 2053 (8.41) |  | 36 (11.96) | |  |
| Activity of daily living: Transfer, No. (% ) |  | 0 (0.00) |  | | 0 (0.00) |
| Independent | 14612 (59.83) |  | 109 (36.21) | |  |
| Needs help | 7041 (28.83) |  | 113 (37.54) | |  |
| Unable | 649 (2.66) |  | 35 (11.63) | |  |
| Can’t evaluate | 1869 (7.65) |  | 41 (13.62) | |  |
| Urinary incontinence, No. (% ) |  | 372 (1.52) |  | | 3 (0.98) |
| Yes | 21533 (88.17) |  | 221 (71.99) | |  |
| No | 2516 (10.30) |  | 83 (27.04) | |  |
| History of fall, No. (% ) |  | 0 (0.00) |  | | 0 (0.00) |
| Yes | 2977 (12.19) |  | 52 (17.28) | |  |
| No | 21004 (86.01) |  | 241 (80.07) | |  |
| Not sure | 440 (1.80) |  | 8 (2.66) | |  |
| Missing | 250 (1.02) |  | 3 (1.00) | |  |
| Index type of admission , No. (% ) |  | 0 (0.00) |  | | 0 (0.00) |
| Via emergency room | 11101 (45.46) |  | 223 (74.09) | |  |
| Direct admission | 365 (1.49) |  | 0 (0.00) | |  |
| Via outpatient department | 12242 (50.13) |  | 73 (24.25) | |  |
| Referral from primary care services | 713 (2.92) |  | 5 (1.66) | |  |
| Health education categories, No. (% ) | 2.31 (2.11) | 0 (0.0) | 3.32 (2.85) | | 0 (0.0) |
| Calcium, mean (SD), mg/dL | 8.91 (0.64) | 15394 (63.04) | 8.78 (0.67) | 166 (54.07) | |
| Creatinine, mean (SD), mg/dL | 1.17 (1.68) | 3489 (14.29) | 1.61 (2.25) | 17 (5.54) | |
| Total bilirubin, mean (SD), mg/dL | 1.03 (1.55) | 19597 (80.25) | 1.52 (2.33) | 181 (58.96) | |
| Aspartate aminotransferase, mean (SD), U/L | 38.13 (51.82) | 10345 (42.36) | 51.76 (75.42) | 117 (38.11) | |
| Alanine aminotransferase, mean (SD), U/L | 30.39 (64.69) | 4210 (17.24) | 37.01 (69.18) | 16 (5.21) | |
| γ-Glutamyltransferase, mean (SD), U/L | 183.29 (225.52) | 23765 (97.34) | 241.21 (373.54) | 293 (95.44) | |
| Lactate dehydrogenase, mean (SD), U/L | 456.42 (1390.08) | 23258 (95.24) | 290.77 (148.40) | 285 (92.83) | |
| Albumin, mean (SD), g/dL | 3.79 (0.73) | 18276 (74.84) | 3.36 (0.70) | 184 (59.93) | |
| C reactive protein, mean (SD), mg/L | 42.48 (58.31) | 20566 (84.21) | 51.12 (66.53) | 218 (71.01) | |
| White Blood Cell, mean (SD), x10^3^ /μL | 7.99 (3.74) | 2398 (9.82) | 8.37 (3.71) | 4 (1.30) | |
| Red Blood Cell, mean (SD), x10^3^ /μL | 4.23 (0.76) | 7233 (29.62) | 3.78 (0.77) | 81 (26.38) | |
| Hemoglobin, mean (SD), g/dL | 12.35 (2.18) | 2086 (8.54) | 11.20 (2.09) | 4 (1.30) | |
| Mean Corpuscular Volume, fL | 88.21 (8.02) | 7420 (30.38) | 89.58 (8.52) | 81 (26.38) | |
| Hematocrit, mean (SD), % | 36.44 (6.35) | 6317 (25.87) | 33.14 (6.27) | 70 (22.80) | |
| Platelet, mean (SD), x10^3^ /μL | 241.12 (93.23) | 2385 (9.77) | 230.86 (104.56) | 5 (1.63) | |
| Thyroid-stimulating hormone, mean (SD), uU/ml | 4.05 (17.33) | 22357 (91.55) | 5.48 (10.71) | 271 (88.27) | |
| Prothrombin time, mean (SD), INR | 1.08 (0.19) | 5788 (23.70) | 1.18 (0.42) | 75 (24.43) | |
| Blood urea nitrogen, mean (SD), mg/dL | 20.44 (17.08) | 7233 (29.62) | 27.94 (26.87) | 69 (22.48) | |
| Sodium, mean (SD), mmole/L | 138.16 (7.10) | 3402 (13.93) | 135.80 (11.30) | 10 (3.26) | |
| Potassium, mean (SD), mmole/L | 3.90 (0.76) | 3356 (13.74) | 3.89 (0.46) | 9 (2.93) | |
| Medical department, No. (% ) | 0 (0.0) |  | 0 (0.0) |  | |
| Internal medicine | 9794 (40.10) |  | 186 (61.79) |  | |
| Surgery | 14627 (59.90) |  | 115 (38.21) |  | |
| Physician’s experience, mean (SD), year | 9.41 (8.02) | 2 (0.01) | 8.51 (7.33) | 0 (0.00) | |
| Discharge planning services, No. (% ) |  | 0 (0.00) |  | 0 (0.00) | |
| Yes | 4856 (19.88) |  | 121 (40.20) |  | |
| No | 19569 (80.12) |  | 180 (59.80) |  | |
| Vital signs in 24 hours prior to discharge, mean (SD) |  |  |  |  | |
| Systolic blood pressure, mmHg | 129.20 (18.66) | 82 (0.34) | 127.38 (19.70) | 0 (0.00) | |
| Diastolic blood pressure, mmHg | 76.05 (12.08) | 83 (0.34) | 73.29 (13.45) | 0 (0.00) | |
| Pulse rate, /min | 78.75 (13.56) | 82 (0.34) | 83.26 (14.24) | 0 (0.00) | |
| Temperature, Celsius | 36.45 (0.62) | 0 (0.00) | 36.50 (0.49) | 0 (0.00) | |
| Respiratory rate, /min | 17.83 (3.28) | 81 (0.33) | 18.41 (2.29) | 0 (0.00) | |
| Destination of discharge, No. (% ) |  | 0 (0.00) |  | 0 (0.00) | |
| Home and outpatient follow up | 23686 (96.99) |  | 299 (97.39) |  | |
| Post-acute care | 162 (0.66) |  | 1 (0.33) |  | |
| Discharge without follow up | 141 (0.58) |  | 1 (0.33) |  | |
| Admitted to other hospital | 346 (1.42) |  | 5 (1.63) |  | |
| Follow up at other hospital | 20 (0.08) |  | 0 (0.00) |  | |
| Home care | 5 (0.02) |  | 0 (0.00) |  | |
| Institutional care | 60 (0.25) |  | 1 (0.33) |  | |
| Others | 1 (0.00) |  | 0 (0.00) |  | |
| No. of discharge drug categories, mean (SD) | 4.45 (3.31) | 0 (0.00) | 6.96 (4.37) | 0 (0.00) | |
| No. of discharge drug tablets, mean (SD) | 56.75 (50.62) | 0 (0.00) | 81.61 (54.28) | 17 (5.54) | |
| Length of stay, mean (SD), days | 6.70 (10.39) | 0 (0.00) | 11.23 (13.11) | 0 (0.00) | |
| Number of catheters, mean (SD) | 0.53 (0.78) | 0 (0.0) | 0.72 (0.91) | 0 (0.0) | |
| Discharge with pressure injury (ies) , No. (% ) |  | 17 (0.07) |  | 0 (0.00) | |
| Yes | 1623 (6.65) |  | 69 (22.92) |  | |
| No | 22799 (93.35) |  | 232 (77.08) |  | |

No.: number; MUST: Malnutrition Universal Screening Tool; BSRS: Brief Symptom Rating Scale
