# Supplementary material for: Predictive modeling for 14-day unplanned hospital readmission risk by using machine learning algorithms
Source: BMC Med Inform Decis Mak. 2021 Oct 20;21:288. doi: 10.1186/s12911-021-01639-y (PMC8527795; doi:10.1186/s12911-021-01639-y)
Supplement: Supplementary file 2 — Additional file 2. The Variance Inflation Factor Values of 27 Features Included in the Final Logistic Regression Model. [file 12911_2021_1639_MOESM2_ESM.docx]

**Additional file 2. The Final Variance Inflation Factor Values of 27 Features Included in the Logistic Regression Model**

| Order | Feature | VIF value |  | Order | Feature | VIF value |
| --- | --- | --- | --- | --- | --- | --- |
| 1 | Education | 3.96 |  | 15 | Risk of fall | 1.98 |
| 2 | C-reactive protein | 3.91 |  | 16 | Length of stay | 1.94 |
| 3 | Marial status | 3.82 |  | 17 | Discharge destination | 1.85 |
| 4 | No. of outpatient visits | 3.26 |  | 18 | Mood | 1.81 |
| 5 | Health education | 3.17 |  | 19 | Creatinine | 1.76 |
| 6 | Total Bilirubin | 3.16 |  | 20 | Thyroid-Stimulating Hormone | 1.54 |
| 7 | Total count of inpatient diagnoses in the past year | 3.05 |  | 21 | Discharge with any catheter | 1.53 |
| 8 | Lactate Dehydrogenase | 2.96 |  | 22 | Charlson comorbidity index | 1.42 |
| 9 | No. of hospitalizations | 2.65 |  | 23 | Nutrition status | 1.40 |
| 10 | Total count of outpatient diagnoses in the past year | 2.55 |  | 24 | Religion | 1.36 |
| 11 | Total number of tablets in discharge medication | 2.51 |  | 25 | Alanine Aminotransferase | 1.34 |
| 12 | Attending physician’s years of experience | 2.36 |  | 26 | Discharge with pressure injury (or injuries) | 1.27 |
| 13 | Gender | 2.01 |  | 27 | History of Fall | 1.23 |
| 14 | No. of emergency department visits | 1.99 |  |  |  |  |
